# Object-Location Memory Training in Older Adults Leads to Greater Deactivation of the Dorsal Default Mode Network

**DOI:** 10.3389/fnhum.2021.623766

**Published:** 2021-02-26

**Authors:** Ania Mikos, Brigitta Malagurski, Franziskus Liem, Susan Mérillat, Lutz Jäncke

**Affiliations:** ^1^University Research Priority Program “Dynamics of Healthy Aging”, University of Zurich, Zurich, Switzerland; ^2^Division of Neuropsychology, Institute of Psychology, University of Zurich, Zurich, Switzerland

**Keywords:** healthy aging, cognitive training, task-based fMRI, default mode network, object-location memory

## Abstract

Substantial evidence indicates that cognitive training can be efficacious for older adults, but findings regarding training-related brain plasticity have been mixed and vary depending on the imaging modality. Recent years have seen a growth in recognition of the importance of large-scale brain networks on cognition. In particular, task-induced deactivation within the default mode network (DMN) is thought to facilitate externally directed cognition, while aging-related decrements in this neural process are related to reduced cognitive performance. It is not yet clear whether task-induced deactivation within the DMN can be enhanced by cognitive training in the elderly. We previously reported durable cognitive improvements in a sample of healthy older adults (age range = 60–75) who completed 6 weeks of process-based object-location memory training (*N* = 36) compared to an active control training group (*N* = 31). The primary aim of the current study is to evaluate whether these cognitive gains are accompanied by training-related changes in task-related DMN deactivation. Given the evidence for heterogeneity of the DMN, we examine task-related activation/deactivation within two separate DMN branches, a ventral branch related to episodic memory and a dorsal branch more closely resembling the canonical DMN. Participants underwent functional magnetic resonance imaging (fMRI) while performing an untrained object-location memory task at four time points before, during, and after the training period. Task-induced (de)activation values were extracted for the ventral and dorsal DMN branches at each time point. Relative to visual fixation baseline: (i) the dorsal DMN was deactivated during the scanner task, while the ventral DMN was activated; (ii) the object-location memory training group exhibited an increase in dorsal DMN deactivation relative to the active control group over the course of training and follow-up; (iii) changes in dorsal DMN deactivation did not correlate with task improvement. These results indicate a training-related enhancement of task-induced deactivation of the dorsal DMN, although the specificity of this improvement to the cognitive task performed in the scanner is not clear.

## Introduction

While normal aging is accompanied by cognitive declines in processing speed, working memory, episodic memory, and reasoning (Park et al., 2002; Salthouse, 2009), promising evidence indicates that the cognitive system demonstrates plasticity across the entire life span (Hertzog et al., 2008) and that cognitive training can improve performance in many of these domains in older adults (Karbach and Verhaeghen, 2014; Chiu et al., 2017). A previously published randomized controlled cognitive training trial from our laboratory targeted an episodic memory process involving the formation of object-location associations (i.e., object-location memory; OLM), which is significantly impaired in old age (Kessels et al., 2007; Old and Naveh-Benjamin, 2008). The OLM training was designed to be process-based, targeting the efficiency of the basic cognitive processes involved through repeated practice (Lövdén et al., 2010). In comparison to an active control training, OLM training led to improvements in the trained task as well as transfer to the domains of spatial memory and reasoning that were maintained 4 months after training (Zimmermann et al., 2016). In the present study, we evaluate whether these training-related behavioral gains were accompanied by changes in neural activity in an important large-scale network (i.e., default mode network, DMN).

While the evidence for training-related improvements in brain structure (e.g., white matter integrity, cortical thickness) is rather limited in older adults (Boyke et al., 2008; Engvig et al., 2012, 2010; Lövdén et al., 2012), changes in neural activity have been more commonly observed (Park and Bischof, 2013). Functional magnetic resonance imaging (fMRI) studies have demonstrated both increases and decreases following cognitive training in task-related activity, mostly in fronto-parietal brain areas (Duda and Sweet, 2019; van Balkom et al., 2020). Reduced activations following training have been interpreted as reflecting increased neural efficiency (Brehmer et al., 2011; Heinzel et al., 2016, 2014), while increased activation can be considered facilitative in older adults who need to overcome age-related neural deficits (Goh and Park, 2009). Further neuroplastic effects have been observed in large-scale brain networks, functionally connected networks of discrete brain regions that are increasingly thought to be essential for successful cognition (Seeley et al., 2007; Wig, 2017). In healthy elderly, cognitive training has been associated with increased intra-network connectivity in the DMN, frontoparietal network (FPN), and salience network (Cao et al., 2016; De Marco et al., 2016), and with increased anti-correlation between the task-negative DMN and the task-positive FPN (Cao et al., 2016; Lebedev et al., 2018).

The DMN, the most widely studied of the large-scale brain networks, demonstrates elevated activity during undirected passive tasks and reduced activity across a wide range of external cognitive tasks (Shulman et al., 1997; Binder et al., 1999; Mazoyer et al., 2001; Buckner et al., 2008). DMN regions, including the posterior cingulate cortex, medial prefrontal cortex, inferior parietal lobule, and medial and lateral temporal lobes, form an intrinsic connectivity network at rest (Greicius et al., 2003) which is mirrored by direct structural connections (Greicius et al., 2009). DMN activation has been largely linked to self-relevant, internally directed information processing (Andrews-Hanna et al., 2010a), including autobiographical memory, self-reflective thought (Gusnard et al., 2001), envisioning future events, mind wandering (Mason et al., 2007), and considering the thoughts and perspectives of others (Raichle et al., 2001; Raichle and Snyder, 2007; Buckner et al., 2008). DMN suppression during specific goal-directed behaviors (i.e., task-induced deactivation, TID) can be interpreted as suspension of this default mode activity (Raichle et al., 2001). TID is positively associated with performance on a wide range of cognitive tasks and increases with task difficulty or cognitive load (McKiernan et al., 2003; Anticevic et al., 2012). Thus, TID is thought to facilitate performance on external cognitive tasks by diverting resources from unconstrained, distracting default mode processes (Binder et al., 1999; Andrews-Hanna, 2012; Anticevic et al., 2012).

In healthy aging, the DMN demonstrates reduced resting state activity and within-network connectivity (Andrews-Hanna et al., 2007; Damoiseaux et al., 2008; Koch et al., 2010; Vidal-Piñeiro et al., 2014; Staffaroni et al., 2018), as well as reduced task-induced deactivation. Compared to younger adults, older adults demonstrate reduced TID within the DMN in tasks of semantic classification (Lustig et al., 2003), memory (Grady et al., 2006), working memory (Sambataro et al., 2010), and visuospatial planning (Spreng and Schacter, 2012). These age-related reductions in TID have been associated with slower performance on tasks of working memory (Brown et al., 2018) and spatial judgment (Park et al., 2010), and worse performance on tests of face-name associative memory (Miller et al., 2008) and verb generation (Persson et al., 2007). Reduced TID is associated with subclinical cognitive decline even in middle-age, suggesting that it may be an early marker for subtle cognitive decline (Hansen et al., 2014).

In the present manuscript, we investigate whether the DMN demonstrates training-related plasticity in healthy older adults, particularly in terms of its capacity for TID. However, the DMN may not deactivate uniformly in response to external cognitive tasks. Growing evidence from detailed high-resolution analyses of single individuals calls into question the idea of a unitary canonical DMN that was historically defined based on group-averaged data, instead suggesting several interwoven networks (Buckner and DiNicola, 2019). Several proposed fractionations have been identified in the resting state literature (i.e., anterior vs. posterior; ventral vs. dorsal) (Damoiseaux et al., 2008; Andrews-Hanna et al., 2010b; Chen et al., 2017). In the task-based domain, Mayer et al. (2010) distinguished between “core DMN” regions that deactivate indiscriminately in response to cognitive demand and other subregions whose deactivation depends on the specific task. Several schemes point to a function involving episodic memory. Andrews-Hanna et al. (2010b) identified a medial temporal lobe subsystem involved in memory-based reconstruction, while the ventral DMN (retrosplenial cortex/medial temporal lobe intrinsic connectivity network) identified in the Shirer atlas (Shirer et al., 2012) exhibited increased functional connectivity during subject-driven episodic memory recall compared to a rest state. Here, we examine task-related activity during performance of an untrained OLM task in the two DMN subnetworks from Shirer et al. (2012): the ventral DMN (vDMN), associated with episodic memory, and the dorsal DMN (dDMN), which more closely resembles the canonical DMN.

In summary, the current study employs task-based fMRI to further elucidate properties of two DMN branches in the context of a randomized controlled cognitive training study, which previously demonstrated improved training-related task performance and durable cognitive transfer effects. The primary aims of the present study are: (i) to investigate the patterns of activation/deactivation within the ventral and dorsal DMN networks during performance on an OLM task at baseline before initiation of training (cross-sectional analysis), (ii) to examine whether the two subnetworks respond to OLM training compared to active control training over the course of several assessments (longitudinal analysis), and (iii) to assess whether any training-related (de)activation changes correlate with improvements in scanner task performance.

## Materials and Methods

### Screening and Training

Participants were recruited at lectures for senior citizens at the University of Zurich, through newspaper articles, advertisements in magazines, public talks, flyers, and word of mouth. All participants gave written informed consent. Inclusion criteria were age between 60 and 75 years, right-handedness, native German speaker or fluent in German, basic computer and internet experience, and access to a computer as well as the internet during the training period. Exclusion criteria were history of previous or current neurological and psychiatric disorders or substance use negatively affecting brain function, sensory and motor deficiencies hindering conduction of training and outcome measurements, violation of MRI safety requirements, and participation in a training study within the last 5 years. In addition, participants who scored 1.5 SD below age-, gender-, and education-specific norms in more than one subtest of the Consortium to Establish a Registry for Alzheimer’s Disease Neuropsychological Assessment Battery (CERAD-NAB; Berres et al., 2000) or had a sum score >5 in the short version of the Geriatric Depression Scale (GDS; Sheikh and Yesavage, 1986) were excluded. Participants were informed about the risks of participation in MRI studies via an information form that they were asked to sign.

After screening, 67 participants were randomized to the object-location memory training condition (OLM group; *N* = 36; mean age = 66.75 ± 4.17) or a control training condition targeting visual perception (active control group, *N* = 31; mean age = 68.23 ± 3.84). There were no baseline differences between groups on demographic or cognitive screening measures (Zimmermann et al., 2016).

Object-location memory and active control training comprised two phases with 15 sessions each that participants had to complete within 3 weeks. A 1-week break separated the phases. Participants trained at home on their personal computers, with each session lasting 30–45 min. Participants were informed that the training software permitted the completion of only one session per day and that they would be contacted by e-mail or phone in case of no recorded training sessions on three consecutive days.

As described in Zimmermann et al. (2016), OLM training consisted of object-location, shape-location, and landmark-location tasks in which cued recall for associations was practiced. Each trial consisted of an encoding phase in which *N* associations had to be encoded, a 20-s distractor task, and a retrieval phase. Task difficulty was adapted to individual performance by increasing or decreasing *N* of to-be-encoded associations by one. Participants started the first session on the lowest level of difficulty with two item-location associations. The highest possible level of difficulty consisted of 21 item-location associations. Individual performance was assessed for the object-, shape-, and landmark-location tasks separately. Task difficulty was increased in the next training session if performance was greater than 70% and was decreased if performance was below 50%. Feedback was given on the percentage of correctly recalled associations and the level of difficulty achieved.

Stimuli for each of the three training tasks were as follows. For the *object-location task, N* objects, drawn from a database of 245 colored drawings of everyday objects (Snodgrass and Vanderwart, 1980; Rossion and Pourtois, 2004), were presented sequentially in a 5-×-6 grid for 4 s followed by an ISI of 0.5 s. For the *shape-location task*, *N* shapes, drawn from a set of 29 self-created shapes in nine different colors were presented for *N* × 3 s. For the *landmark-location task*, stimuli were drawn from a database of 261 photographs of real-world buildings (retrieved from the internet, excluding highly salient or famous buildings) and presented on a 6-×-6 grid with a different self-created city map superimposed in each training session. During the retrieval phase, the previously presented stimuli were displayed along with the empty grid and participants had to select the cell in which the stimulus was initially presented with a mouse click.

Active control training included two phases of a visual perception task, separated by a distractor task. Stimuli and task duration were matched to those of the OLM training tasks. For the *object-perception task*, two 1-×-10 grids filled with objects were presented, and participants had to click with the mouse on the one object that differed between the two grids. For the *shape-perception task*, participants selected a target shape within a 6-×-6 grid filled with 36 shapes. For the *landmark-perception task*, participants selected a target building from within a city map filled with 21 buildings.

Five participants completed only 27–29 training sessions [OLM group: 29 sessions (*N* = 1), 27 sessions (*N* = 1); active control group: 29 sessions (*N* = 2), 28 sessions (*N* = 1)] because of technical and scheduling problems. Two participants from the OLM group completed one additional training session (in the second training phase: *N* = 1, in the follow-up period: *N* = 1).

### Scanner Task

Both groups underwent fMRI while performing an object-location memory task during four sessions: baseline before initiation of either training condition (T1), after the first 3-week training phase (T2), at the conclusion of the 6-week training period (T3), and 4 months after training completion (T4). A block design was used, consisting of 24 blocks equally divided between two runs. Each run lasted approximately 14 min. The order of the two runs was counterbalanced across participants of both groups. Before entering the scanner, participants completed five practice trials of the task on a laptop. In the MR scanner, participants lay comfortably in supine position with padded head holders restricting head movements. Stimuli were presented using the software Presentation (NeuroBehavioral Systems; NBS) which also recorded behavioral performance. The stimuli were presented on MR compatible goggles (Resonance Technology Inc., Northridge, CA, United States) which could be adjusted for poor eyesight. Object stimuli were drawn from the Bank of Standardized Stimuli (BOSS) created by Brodeur et al. (2010). For the present study, the 480 colored everyday objects were divided into two sets with comparable familiarity and object identity ratings (provided by Brodeur et al., 2010). After eliminating photo stimuli which were semantically very similar (i.e., wire, cable) and stimuli that were mostly white in color because of the white screen background, 144 stimuli for each fMRI run remained. The same number of similarly rated stimuli was used for each encoding phase. Stimuli were used only once within both runs. They were different from the object stimuli used for the OLM and active control training tasks.

A block of the scanner OLM task included four phases: encoding, distractor, recognition, and visual fixation baseline. Stimuli were presented in a 5 × 5-grid on a white background. During the encoding phase, six objects were presented serially in one of the grid cells, each for 3000 ms (ISI = 0 ms). During the distractor phase, participants were asked to solve a 1-back task. Black arrows were presented consecutively in random order and participants had to decide whether the presented arrow pointed toward the same direction as the previous one and to indicate their decisions by pressing one of two buttons on an MR compatible response box with their left or right thumbs (same direction = left, different direction = right). Each cue lasted for 1000 ms followed by an ISI of 1000 ms. The distractor phase was randomly jittered and lasted for 12000–18000 ms. During the recognition phase, participants were presented the six encoded objects sequentially in the 5 × 5-grid, each for 3000 ms (ISI = 0 ms). Three of the objects appeared in the same locations as during encoding, whereas three objects were presented in locations in which different objects had been displayed during encoding. Participants had to decide whether presented object-location associations were the encoded ones or not and indicate their decisions by pressing one of two buttons on the response box with their left or right thumbs. The subsequent visual fixation baseline phase was jittered and lasted between 9000 and 15000 ms. During this period, a black cross was presented that changed to green 2000 ms before the encoding phase of the next block started.

**FIGURE 1:**
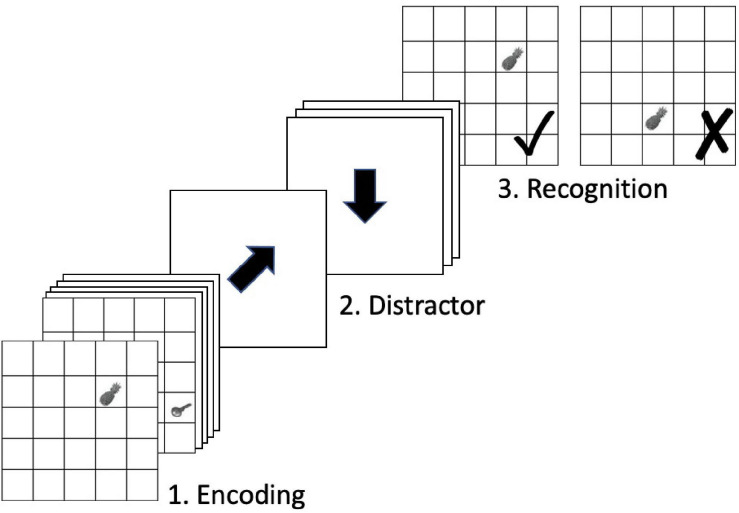
In-scanner OLM task. Figure adapted from Zimmermann (2015).

The scanner task is displayed in Figure 1. The behavioral dependent variables of interest include *hits*, defined as the number of correct responses out of 72 for each run. Hits were averaged across the two runs for each time point, yielding the variable *average hits* used in the following analyses. Additionally, reaction time (in milliseconds) was averaged across hit trials only and then across runs to provide *hit reaction time*.

Scanner task average hits contributed to the near-transfer spatial episodic memory composite outcome measure of this trial, reported in Zimmermann et al. (2016). In this regard, it is important to note that although the scanner task and OLM training task were somewhat similar, they differed in several important ways. Object stimuli for the scanner and training tasks were drawn from two different sources, and the training task additionally included landmark and shape stimuli. Further, the two tasks differed in the rate of stimulus presentation during encoding, duration and content of the distractor phase, and task demands during retrieval. As such, scanner task average hits is thought to represent near-transfer effects rather than training effects *per se*.

### MRI Protocol

Whole brain T2-weighted EPI-BOLD data were acquired with a Philips Achieva 3T TX scanner (Philips Medical Systems, Best, Netherlands) using a 32-channel receiver head coil array. Blood-oxygen-level-dependent (BOLD) fMRI images were generated with a gradient-echo-planar-imaging (EPI) pulse sequence (TR/TE = 2500/30 ms, flip angle = 84°, matrix = 80 × 80, FOV = 240 mm × 240 mm, 44 slices, slice thickness 3 mm, 0.5 mm interslice spacing), that yielded 3 mm × 3 mm × 3 mm voxels. Slices were acquired in descending order and in transverse orientation. Each of the two runs consisted of a total of 335 volumes. Five dummy scans were performed prior to image acquisition aiming to eliminate signals arising from progressive saturation. In addition, a high-resolution T1 anatomic image (TR/TE = 8.1/3.7 ms, flip angle = 8°, matrix 240 × 240, FOV = 240 mm × 240 mm, 160 slices, slice thickness 1.0 mm, 1 mm × 1 mm × 1 mm voxels) was obtained for each subject.

### Functional Image Analyses

Imaging data were first transformed into the Brain Imaging Data Structure (BIDS) format (Gorgolewski et al., 2016). Preprocessing of each functional run was performed using fMRIPrep version 1.2.6 (Esteban et al., 2018), a Nipype (Gorgolewski et al., 2011) based tool, including the following steps: bias field correction, skull stripping, correction for head-motion parameters, slice time correction, co-registration to corresponding structural image [boundary-based registration with 9 degrees of freedom implemented in FreeSurfer v6.0.0 (Greve and Fischl, 2009)], and spatial normalization to MNI space. Motion correcting transformations, T1 weighted transformation and MNI template warp were applied in a single step using antsApplyTransformations v2.1.0 with Lanczos interpolation. Three tissue classes were extracted from T1 images using FSL FAST v5.0.9 (Zhang et al., 2001). Voxels from cerebrospinal fluid and white matter were used to create a mask in turn used to extract physiological noise regressors using aCompCor (Behzadi et al., 2007). The mask was eroded and limited to subcortical regions to limit overlap with gray matter; six principal components were estimated. Framewise displacement (Power et al., 2014) was calculated for each functional run using Nipype implementation. For more details of the pipeline see: http://fmriprep.readthedocs.io/en/latest/workflows.html.

**FIGURE 2:**
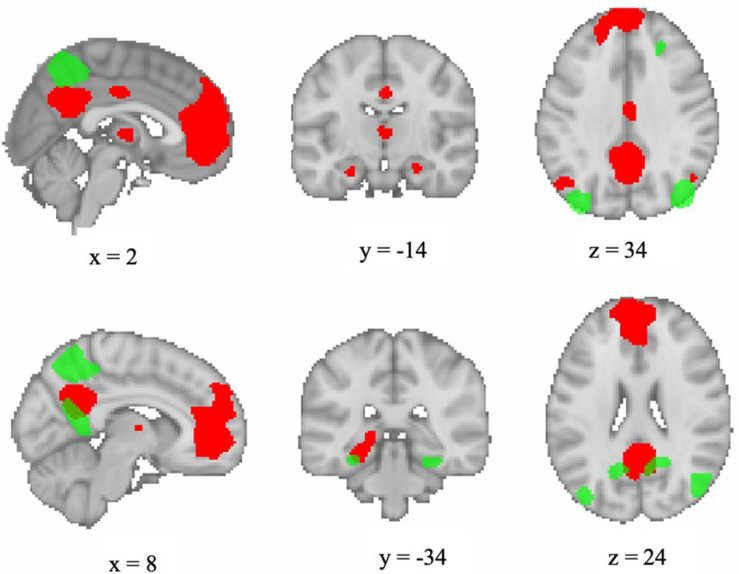
Visualization of functional ROIs comprising dorsal DMN (red) and ventral DMN (green) networks as defined by Shirer et al. (2012). Image generated in FSLeyes version 0.27.0 (https://git.fmrib.ox.ac.uk/fsl/fsleyes/fsleyes/) using a template brain and displayed in neurological orientation.

First-level statistical analyses were performed using the general linear model approach (GLM) as implemented in SPM12^1^. Explanatory variables modeling the experimental conditions of the blocked fMRI design comprised the following four conditions: (1) encoding, (2) distractor, (3) recognition, and (4) visual fixation baseline. These four conditions were modeled for each of the two fMRI runs at each session (T1, T2, T3, and T4). In addition, the GLM included the six motion parameters, the framewise displacement, and physiological noise regressors [first principal component from aCompCor (Behzadi et al., 2007)] as obtained from fMRIPrep preprocessing in order to control for physiological and movement confounds. Contrast images were created for: (1) encoding vs. visual fixation baseline and (2) recognition vs. visual fixation baseline. The MarsBaR SPM toolbox (v0.44, marsbar.sourceforge.net) was used to extract contrast values from DMN networks for each run. Regions of interest (ROIs) for the DMN networks were selected from the atlas of Shirer et al. (2012). The atlas comprises 90 functionally derived ROIs across 14 intrinsic connectivity networks, identified via independent component analysis. Functional ROIs were shown to outperform a set of commonly used structural ROIs in classifying subject-driven cognitive states, and patterns of within- and between-network functional connectivity were related to subject-driven cognitive states (Shirer et al., 2012). For the present study, contrast values were extracted for each of the dorsal and ventral default mode networks; Figure 2 displays the ROIs comprising each network. Each participants’ extracted coefficient estimates were averaged across the two runs of each assessment time and then submitted to second-level analysis.

### Missing Data

fMRI assessments were completed by all 67 participants with the exception of two participants (one from each group) who did not take part in T4 testing because of medical reasons. Three participants from the control group only completed one of the two runs at T4, two for technical reasons and one for medical reasons. For these three participants, data from the one completed run at T4 were used in statistical analyses.

### Statistical Analyses

Scanner task data (average hits and hit reaction time) were first screened for outliers. Excessive values were defined as any value more than three median absolute deviations (MADs) above the median of the sample distribution for each group at each measurement occasion (Leys et al., 2013). Very few outliers were detected (average hits: 1 outlier from each group; hit reaction time: 1 participant from the active control group produced outlier values at T3 and T4). Removal of these outlier values did not change the overall pattern of results; therefore, analysis results from the complete data set are reported below.

For baseline (T1) analyses, we conducted independent samples *t*-tests to evaluate whether there were group differences on the scanner task or network (de)activation levels. Network (de)activation levels were also compared between the encoding and recognition conditions using independent samples *t*-tests.

For longitudinal analyses, linear mixed effects regression (LMER) was used to determine if there were statistically reliable differences for behavioral performance on the scanner task as well as for levels of network (de)activation. Three pairs of models were conducted: one pair of behavioral models with dependent variables of average hits and hit reaction time on the scanner task, and two pairs of network models (one for the dDMN and one for the vDMN), each with dependent variables of (de)activation levels in each of two task conditions (encoding and recognition). Fixed-effects predictors included group (OLM vs. active control) and time of assessment, coded as a factor including levels T2, T3, and T4. Baseline (T1) levels of the behavioral variable (for the behavioral models) or network (de)activation level (for the network models) were included as covariates. Age and sex were also included as covariates in all models. Participant was modeled as a random effect to account for random variability between individuals. For all LMER models, α was set at 0.025 in order to incorporate a Bonferroni correction reflecting the fact that there were two behavioral models and two models for each of the DMN networks.

All analyses were performed in R version 3.6.0 (R Core Team., 2019). The *Routliers* package (Delacre and Klein, 2019) was used to detect outliers using the Median Absolute Deviation (Leys et al., 2013). LMER models were conducted using the *lme4* package (Bates et al., 2015) with restricted maximal likelihood estimation. Degrees of freedom for the fixed effects were estimated by the Satterthwaite approximation as implemented in the package *lmerTest* (Kuznetsova et al., 2017). The package *emmeans* (Lenth, 2020) was used to evaluate significance of marginal contrasts for training group (OLM versus active control), sex, time (each time point contrasted against the previous time point), and group X time interaction (group contrast evaluated separately at T2, T3, and T4). Simple approximations of between-subjects effect sizes reported as Cohen’s *d* (small = 0.2; medium = 0.5; large = 0.8) were produced with the *effectsize* package (Ben-Shachar et al., 2020) using the estimated *t* statistic and observed degrees of freedom. The package *ggplot2* (Wickham, 2016) was used to make the figures.

## Results

For visualization purposes, mean scanner task performance and DMN subnetwork (de)activation levels across all four time points for each group are displayed in Supplementary Figures 1, 2. The following analyses first address baseline (T1) results and then examine whether training effects can be detected in a longitudinal framework.

### Baseline Values and Group Comparability

#### Baseline Scanner Task Performance

We first verified baseline comparability of behavioral results across groups. Mean baseline values for scanner task outcomes and results of independent samples *t*-tests comparing the two groups are displayed in Table 1. There were no significant differences between groups for average hits or hit reaction time on the scanner task at T1.

**TABLE 1:** Descriptive statistics and results of independent-samples *t*-tests for baseline scanner task performance.

	OLM group		Active control group				
Dependent variable	Mean	*SD*	Mean	*SD*	*t*	*p*	*d*
Average hits^a^	57.03	4.71	57.44	5.21	−0.34	0.738	−0.08
Hit reaction time (ms)	1292.88	159.96	1240.40	148.53	1.38	0.171	0.34

*^*a*^Refers to the number of correct responses on the scanner task out of 72 items for each run, averaged across two runs.*

#### Baseline DMN Subnetwork Results

**FIGURE 3:**
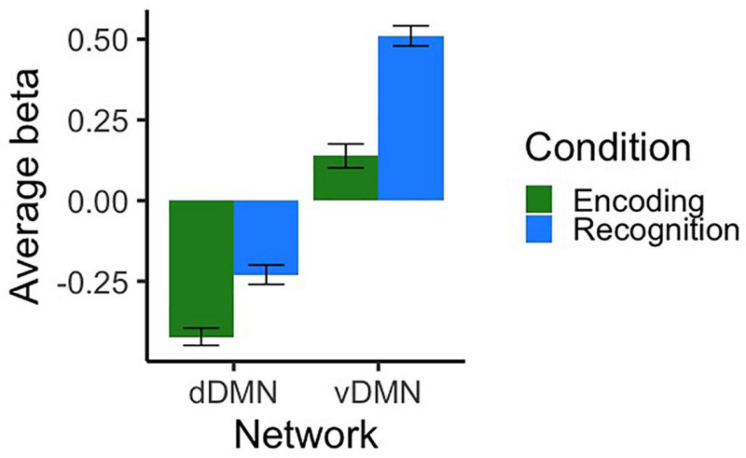
Average beta values representing activation/deactivation at T1 for dorsal and ventral DMN networks during both conditions of the scanner task. Contrasts represent encoding and recognition conditions relative to the visual fixation baseline condition. Error bars represent SEM.

Cross-sectional fMRI results from T1 were evaluated in order to characterize baseline patterns of activation across networks/conditions and to determine whether there were any baseline differences by group, age, or sex. As shown in Figure 3, the dDMN was deactivated during both task conditions and the vDMN was activated. We conducted independent samples *t*-tests to compare encoding and recognition values for each network. For the dDMN, there was more deactivation during encoding than recognition, *t*(132) = −4.78, *p* < 0.001, *d* = −0.83. For the vDMN, there was more activation during recognition than encoding, *t*(132) = −7.69, *p* < 0.001, *d* = −1.34. In summary, the dDMN was deactivated during both conditions of the memory task, more so during encoding, and the vDMN was activated during both conditions, more so during recognition.

Next, we evaluated baseline comparability of DMN subnetwork (de)activation across groups. Mean contrast values and results of independent samples *t*-tests comparing the two groups are displayed in Table 2. Across both networks, there were no significant group differences in contrast values for either task condition. Further regression models were conducted to test if there were effects of age or sex on baseline contrast values. Four separate models were conducted with beta value for dDMN encoding, dDMN recognition, vDMN encoding, and vDMN recognition as the dependent variables. There was no significant effects of age or sex for any of the models (all *p*-values > 0.11). Thus, results suggest that the two groups were comparable in terms of baseline levels of task-related (de)activation, and no age or sex effects were detected.

**TABLE 2:** Descriptive statistics and results of independent-samples *t*-tests for network activity at baseline.

		OLM group		Active control group				
Network	Condition	Mean	SD	Mean	SD	*t*	*p*	*d*
Dorsal DMN	Encoding	−0.41	0.23	−0.44	0.21	0.68	0.501	0.17
	Recognition	−0.20	0.23	−0.26	0.27	1.02	0.309	0.25
Ventral DMN	Encoding	0.12	0.32	0.16	0.28	−0.57	0.571	−0.14
	Recognition	0.56	0.21	0.45	0.29	1.80	0.077	0.45

### Longitudinal Analyses

#### Effect of Training Group on Scanner Task Performance

**FIGURE 4:**
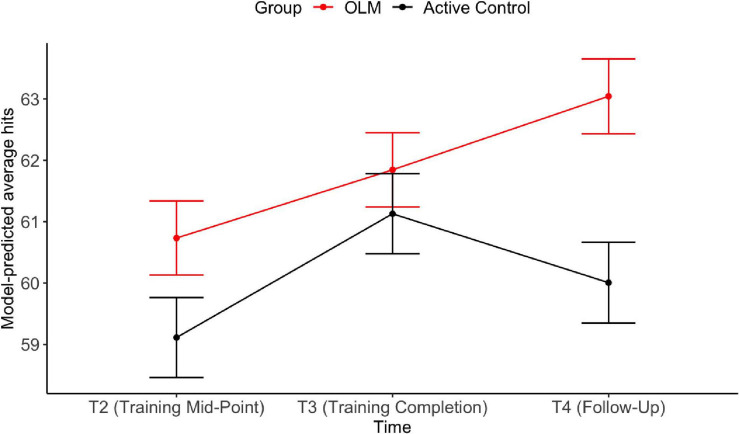
Estimated marginal means from LMER models for average hits during the scanner task. Error bars represent SEM.

The first longitudinal analysis evaluated the impact of training on scanner task performance. Table 3 shows the results of the LMER models evaluating fixed effects of group and time on the behavioral fMRI dependent variables (average hits and hit reaction time on the scanner task). For average hits, the effect of T3 against T2 was significant, indicating improved performance across both groups from T2 to T3. There was a significant main effect of group, whereby the OLM group performed better across T2–T4. Further, there was a significant interaction such that at T4, the OLM group performed significantly better than the control group (see Figure 4). These OLM training effects constituted medium effect sizes (main effect of group: *d* = 0.64, effect of group at T4: *d* = 0.6). There were no significant effects of age or sex on average hits.

**TABLE 3:** Parameter estimates for fixed effects related to the scanner task.

Outcome	Predictor	Estimate	*SE*	*t*	*p*	*d*
Average hits	Sex^a^	−0.66	0.73	−0.90	0.370	−0.23
	Age	−0.04	0.09	−0.40	0.691	
	**T1 average hits**	**0.60**	**0.07**	**8.12**	**<0.001**	
	**Group^b^**	**1.79**	**0.73**	**2.47**	**0.016**	**0.63**
	**T3^c^**	**1.56**	**0.44**	**3.54**	**<0.001**	
	T4^c^	0.04	0.45	0.08	0.93	
	Group^b^ X T2	1.62	0.89	1.83	0.070	0.33
	Group^b^ X T3	0.72	0.89	0.81	0.421	0.14
	**Group^b^X T4**	**3.04**	**0.90**	**3.39**	**<0.001**	**0.60**
Hit RT	Sex^a^	9.78	22.20	0.44	0.662	0.11
	Age	1.02	2.75	0.37	0.711	
	**T1 RT**	**0.78**	**0.07**	**10.64**	**<0.001**	
	Group^b^	−11.40	22.10	−0.52	0.608	−0.13
	**T3^c^**	**−32.30**	**11.80**	**−2.74**	**0.007**	
	T4^c^	1.33	12.00	0.11	0.911	
	Group^b^ X T2	−0.24	25.90	−0.01	0.993	0.00
	Group^b^ X T3	14.42	25.90	0.56	0.580	0.11
	Group^b^ X T4	−48.46	26.30	−1.84	0.068	−0.35

*^*a*^Male contrasted against female.^*b*^OLM training group contrasted against active control group. ^*c*^Contrasted against the preceding time of assessment.*P*-values are uncorrected. Cohen’s d for between-subjects effects based on parameter estimates from linear mixed effects regression models. Statistically significant effects surviving Bonferroni correction appear in bold. RT, reaction time.*

For hit reaction time, the effect of T3 against T2 was significant, indicating that reaction time decreased across both groups from T2 to T3. There was no significant main effect of group and no group X time interactions. There were no significant effects of age or sex on hit reaction time.

#### Effect of Training Group on Network Activation/Deactivation

The next longitudinal analyses evaluated whether there were statistically reliable differences in network (de)activation as a result of OLM training when controlling for baseline (T1) network activity. Performance on the scanner task (average hits) was included as an additional covariate, since behavioral analyses revealed a group difference on this variable.

##### Dorsal DMN

Fixed effects results of the LMER models for the dDMN are summarized in Table 4. For both conditions, baseline deactivation was positively and significantly related to the dependent variable (DV), indicating that T2, T3, and T4 deactivation levels are positively correlated with baseline (T1) deactivation.

**TABLE 4:** Dorsal DMN: Parameter estimates for fixed effects related to activation/deactivation for both conditions of the scanner task.

Condition	Predictor	Estimate	*SE*	*t*	*p*	*d*
Encoding	**Sex^a^**	**−0.12**	**0.05**	**−2.45**	**0.017**	**−0.62**
	Age	0.00	0.01	0.24	0.808	
	**T1 deactivation**	**0.76**	**0.11**	**7.16**	**<0.001**	
	Average hits	0.00	0.00	0.68	0.500	
	**Group^b^**	**−0.12**	**0.05**	**2.42**	**0.019**	**−0.61**
	T3^c^	0.01	0.03	0.32	0.747	
	T4^c^	−0.04	0.03	−1.17	0.245	
	Group^b^ X T2	−0.06	0.06	−1.01	0.315	−0.18
	**Group^b^ X T3**	**−0.14**	**0.06**	**−2.40**	**0.018**	**−0.43**
	**Group^b^ X T4**	**−0.15**	**0.06**	**−2.44**	**0.016**	**−0.43**
Recognition	Sex^a^	−0.04	0.04	−1.02	0.314	−0.26
	Age	0.00	0.01	0.61	0.543	
	**T1 deactivation**	**0.57**	**0.09**	**6.34**	**<0.001**	
	Average hits	0.01	0.00	1.49	0.137	
	Group^b^	−0.06	0.04	−1.29	0.202	−0.33
	T3^c^	0.00	0.03	−0.08	0.939	
	T4^c^	0.02	0.03	0.77	0.442	
	Group^b^ X T2	−0.02	0.05	−0.34	0.738	−0.06
	Group^b^ X T3	−0.10	0.05	−1.95	0.054	−0.36
	Group^b^ X T4	−0.05	0.05	−0.91	0.367	−0.16

*^*a*^Male contrasted against female.^*b*^OLM training group contrasted against active control group. ^*c*^Contrasted against the preceding time of assessment.*P*-values are uncorrected. Cohen’s d for between-subjects effects based on parameter estimates from linear mixed effects regression models. Statistically significant effects surviving Bonferroni correction appear in bold.*

**FIGURE 5:**
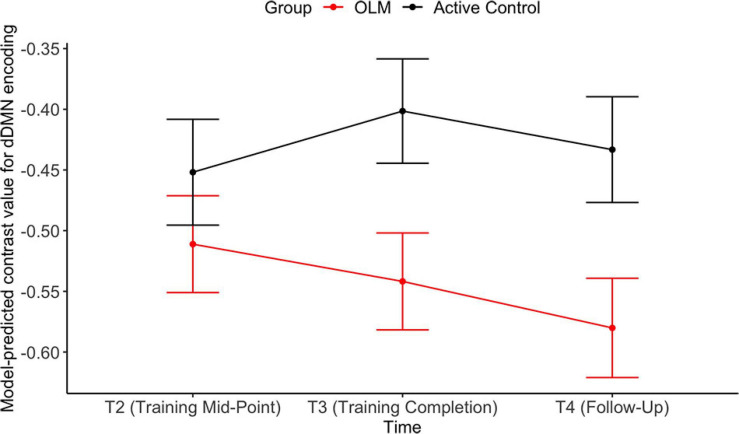
Estimated marginal means representing group X time interactions in the dDMN encoding LMER model. Error bars represent SEM.

The group contrast predictor was significant only in the encoding model, indicating that across T2–T4 the OLM group exhibited more deactivation compared to the active control group (*d* = −0.61). Examination of the group contrast at each time point revealed that there were significant group differences at T3 and T4 (both *d* = −0.43). Estimated marginal means from these group by time interactions are shown in Figure 5. Additionally, there was a significant effect of sex during the encoding condition, with men showing greater dDMN deactivation than women (*d* = −0.62). Because sex and group were both significant in the encoding model, the model was re-run allowing group and sex to interact. However, this interaction was not significant (*p* = 0.85), indicating that the stronger dDMN deactivation in men does not seem to drive the OLM training group effect.

For the recognition model, group was not significant as a main effect, and there were no significant group X time interactions. There was no significant association between dDMN deactivation and scanner task average hits or age in either condition.

##### Ventral DMN

Fixed effects results of the LMER models for the vDMN are summarized in Table 5. For both conditions, baseline level of activation was positively and significantly related to the DV, indicating that T2, T3, and T4 activation levels are positively correlated with baseline (T1) activation.

**TABLE 5:** Ventral DMN: Parameter estimates for fixed effects related to activation/deactivation for both conditions of the scanner task.

Condition	Predictor	Estimate	*SE*	*t*	*p*	*d*
Encoding	Sex^a^	−0.08	0.05	−1.55	0.127	−0.39
	Age	0.01	0.01	1.33	0.190	
	**T1 activation**	**0.67**	**0.08**	**8.02**	**<0.001**	
	Average hits	0.01	0.00	1.78	0.076	
	Group^b^	0.03	0.05	0.61	0.547	0.15
	T3^c,d^	0.07	0.03	2.11	0.036	
	**T4^c^**	**−0.10**	**0.03**	**−2.97**	**0.004**	
	Group^b^ X T2	0.07	0.06	1.19	0.236	0.20
	Group^b^ X T3	0.02	0.06	0.30	0.768	0.05
	Group^b^ X T4	0.00	0.06	−0.06	0.956	−0.01
Recognition	Sex^a^	0.01	0.04	0.37	0.713	0.09
	Age	0.01	0.00	1.30	0.199	
	**T1 activation**	**0.77**	**0.08**	**9.60**	**<0.001**	
	**Average hits**	**0.01**	**0.00**	**2.63**	**0.009**	
	Group^b^	0.05	0.04	1.22	0.226	0.31
	T3^c,d^	0.06	0.03	2.20	0.030	
	**T4^c^**	**−0.08**	**0.03**	**−2.86**	**0.005**	
	Group^b^ X T2	0.07	0.05	1.46	0.147	0.26
	Group^b^ X T3	0.02	0.05	0.38	0.702	0.07
	Group^b^ X T4	0.06	0.05	1.10	0.273	0.19

*^*a*^Male contrasted against female. ^*b*^OLM training group contrasted against active control group. ^*c*^Contrasted against the preceding time of assessment.^*d*^Does not survive multiple comparison corrections (alpha = 0.05/2).*P*-values are uncorrected. Cohen’s *d* for between-subjects effects based on parameter estimates from linear mixed effects regression models. Statistically significant effects surviving Bonferroni correction appear in bold.*

Group was not significant as a main effect for either condition. Effects of time were significant in both the encoding and recognition models. Specifically, when averaging across groups, activation increased from T2 to T3, but these differences did not survive correction for multiple comparisons. Activation then decreased significantly across groups from T3 to T4. There were no significant effects of age or sex in either condition. Finally, scanner task average hits were positively related to vDMN activation during the recognition condition.

##### Exploratory analyses: Dorsal DMN nodes

*Post-hoc* tests were conducted in order to examine whether the OLM-training-related increase in dDMN deactivation during encoding occurred across all constituent nodes or was driven by changes in only a select few. A series of LMER models was conducted with the same fixed effects, covariates, and random effects as for the longitudinal network analyses. This time, the DVs were the encoding contrast values for each of the nine functional ROIs comprising the dDMN (Shirer et al., 2012). The beta values for the group contrast (across T2–T4) from each model are shown in Table 6. The OLM group exhibited significantly greater deactivation in the medial prefrontal cortex, right superior frontal gyrus, and midcingulate cortex compared to the active control group. Only the medial prefrontal cortex and midcingulate cortex group differences survived Bonferroni-Holm correction for multiple comparisons. The significant group contrasts across T2–T4 demonstrated medium to large effect sizes (midcingulate cortex, *d* = −0.72; medial prefrontal cortex, *d* = −0.83).

**TABLE 6:** Dorsal DMN nodes: Parameter estimates for group effect (across T2–T4) in the encoding condition.

Node	Estimate	*SE*	*t*	*p*	*d*
**Medial PFC, OFC**	**−0.16**	**0.05**	**−3.29**	**0.002**	**−0.83**
Left angular gyrus	−0.09	0.11	−0.84	0.402	−0.21
Right superior frontal gyrus^a^	−0.10	0.05	−2.06	0.043	−0.53
PCC, precuneus	−0.03	0.07	−0.43	0.673	−0.11
**Midcingulate cortex**	**−0.22**	**0.08**	**−2.82**	**0.006**	**−0.72**
Right angular gyrus	0.01	0.10	0.06	0.954	0.01
Left and right thalamus	0.00	0.07	0.02	0.986	0.00
Left hippocampus	−0.07	0.06	−1.24	0.219	−0.31
Right hippocampus	−0.13	0.06	−2.21	0.031	−0.56

*^*a*^Does not survive Bonferroni-Holm correction for multiple comparisons. OLM training group is contrasted against active control group. *P*-values are uncorrected. Cohen’s *d* is based on parameter estimates from linear mixed effects regression models. Statistically significant effects surviving multiple comparisons correction appear in bold. PFC, prefrontal cortex; OFC, orbitofrontal cortex; PCC, posterior cingulate cortex.*

#### Head Motion

We evaluated whether group differences existed in the amount of head motion, defined as the average framewise displacement (FD) at each measurement occasion; T1 median = 0.21 (range 0.08–1.04); T2 median = 0.20 (range = 0.09–0.7); T3 median = 0.20 (range = 0.07–0.77); T4 median = 0.22 (range 0.09–0.9). Mann–Whitney *U* tests were conducted given that FD values were not normally distributed. There were no significant group differences in FD at any of the time points (all *p*-values ≥ 0.34), suggesting that group differences in dDMN deactivation cannot be explained by group differences in head motion.

### Neural Correlates of Task Improvement

The results presented above indicate that relative to the active control group, the OLM group demonstrated significantly more scanner task average hits at T4 as well as increased task-induced deactivation of the dDMN during encoding. We therefore evaluated if within-person change in dDMN deactivation levels during encoding (T4–T1) predicted scanner task average hits at T4. We calculated residuals scores for T4 average hits and for the within-person change in deactivation by regressing out the effects of age, sex, T1 average hits, and T1 deactivation values. Next, we examined the correlation between the residualized values for deactivation change and T4 average hits for each group separately. Correlation coefficients were not significant for either group (OLM: *r* = −0.27, *p* = 0.11; active control: *r* = 0.13, *p* = 0.51). Two outliers were detected for the residualized average hits variable within the OLM group; the correlation remained non-significant after removal of these two outliers (*r* = −0.15, *p* = 0.40).

## Discussion

Findings highlight the functional heterogeneity of the DMN both in terms of task-based activation/deactivation during an object-location memory task and its response to cognitive training in healthy older individuals. Specifically, the ventral DMN was activated during the encoding and recognition conditions of the scanner task and the dorsal DMN was deactivated. Further, we report the novel finding that task-induced deactivation within the dorsal DMN was enhanced by process-based cognitive training compared to an active control training condition. Although the functional implications of this finding are not entirely clear, we discuss potential interpretations in terms of current theories of DMN functioning below.

### Task-Related Activation/Deactivation of the Ventral and Dorsal DMN

Activation of the vDMN observed in the baseline (T1) analysis is consistent with the proposed role of a medial temporal lobe DMN subsystems in episodic memory. The vDMN as defined by Shirer et al. (2012) connects known memory regions such as the retrosplenial cortex and medial temporal lobe and demonstrates increased functional connectivity when subjects freely recall events of their day. The medial temporal lobe DMN system is thought to especially relate to associative aspects of memory, such as the retrieval of additional contextual details related to how the item was initially encountered (Andrews-Hanna et al., 2014). Associative memory was an important feature in our in-scanner OLM task paradigm, because subjects needed to retrieve the spatial location of the stimulus from the encoding condition in order to correctly answer the recognition probe. In the longitudinal model, the number of average hits from the scanner task was positively associated with vDMN activation during the recognition condition, and the baseline analysis indicated that vDMN activation was greater during recognition than encoding. These findings support the role of the vDMN particularly during the retrieval of object location associations.

The dorsal DMN, on the other hand, demonstrated task-induced deactivation. As defined in Shirer et al. (2012) on the basis of functional connectivity during subject-driven cognitive states, the dDMN consists of areas including the medial prefrontal cortex, posterior cingulate cortex, precuneus, and angular gyrus. These regions correspond to the classical DMN regions long observed to exhibit task-induced deactivation (Shulman et al., 1997; Raichle et al., 2001). In our baseline analysis, deactivation was greater during the encoding than the recognition condition. Further, the training effect of increased TID within the dorsal DMN was apparent in the encoding condition, and we discuss the potential significance of TID during encoding below.

### Training-Related Increase in Dorsal DMN Deactivation

Object-location memory training was associated with significantly more dDMN deactivation during encoding relative to active control training. Analyses controlled for baseline (T1) deactivation levels, age, sex, and average hits, and thus cannot be explained by differences in these factors. Levels of head motion did not differ between the two groups at any time point, and therefore also do not seem to explain differences in deactivation.

In the following section, we interpret this task-related deactivation in the context of the results of the behavioral task that was performed concurrently in the scanner. The scanner OLM task differed from the training tasks performed by the OLM group, and it is considered to represent near-transfer within the domain of spatial episodic memory. This has implications for interpretation of the behavioral results. For instance, it may be somewhat surprising that the active control group improved to a similar degree as the OLM group from mid-training (T2) to T3 (end of training) such that performance for both groups was equivalent at T3. However, since the scanner task differed from the OLM training task, it may be a less sensitive behavioral outcome measure than the training task itself.

While both groups demonstrated improved average hits performance at T3 relative to T2, only the OLM group showed the neural effect of increased dDMN deactivation at T3. Thus, it appears that the behavioral performance of the active control group improved at T3 but without the underlying neural change that was shown by the OLM group. This suggests that the improvement in each group relative to the previous time point may have involved different underlying mechanisms. Improvement in the active control group could have been due to practice effects and/or the mental engagement afforded by the active control perceptual training tasks without any underlying change in dDMN deactivation. The behavioral effect observed in the OLM group at T3 might reflect an increase in neural capacity, as evidenced by the concurrent increase in deactivation. Further, the OLM group maintained their improved average hits performance at T4, while the active control group did not. It is possible that the underlying neural response, evident at T3 as well as T4 explains the maintenance of the behavioral improvement in the OLM group.

Still, we did not find direct evidence for a relationship between increased dDMN suppression and improved task performance in the OLM group at T4. Lack of a significant brain-behavior relationship does not necessarily prove a lack of functional significance and could be related to several factors, including a lack of power. Further, TID may represent a task-independent phenomenon that does not directly correlate with improvement on this particular task. Indeed, a notable feature of TID is the consistency of deactivated regions across a wide range of cognitive tasks (Shulman et al., 1997; Gusnard and Raichle, 2001). Although some studies have noted a relationship between the degree of deactivation and task performance as measured by reaction time or accuracy (Persson et al., 2007; Miller et al., 2008; Park et al., 2010; Brown et al., 2018), DMN suppression may not directly relate to object-location memory performance *per se* but may instead represent a more general, task-independent phenomenon.

### Relationship to Theories of DMN and TID

The mechanisms underlying TID are not fully understood, but one proposed mechanism reflects a reallocation of limited brain processing resources when attention shifts from ongoing, internal, conceptual processes to performance of an exogenous task (Binder et al., 1999; McKiernan et al., 2003). Consistent with this model, greater TID magnitude during an external task is associated with a lower frequency of task-unrelated thoughts (McKiernan et al., 2006), while reduced TID is related to attentional lapses and errors (Weissman et al., 2006; Li et al., 2007; Eichele et al., 2008). Additionally, activation of the DMN has been associated with mind wandering during a task that was highly practiced in order to elicit stimulus-independent thoughts (Mason et al., 2007) and with mind wandering during meditation (Hasenkamp et al., 2012). Finally, experience sampling during fMRI acquisition revealed that DMN activations preceded off-task thoughts and errors (Christoff et al., 2009). Drawing from this model, we interpret our finding of increased TID in the OLM group as reflecting a greater ability to reallocate processing resources from default mode areas. Behaviorally, this enhanced DMN deactivation may reflect a greater ability to suppress default cognitive processes, such as mind wandering, task-unrelated thoughts, and self-referential processing, in order to focus on the external task.

Although the role of DMN activation in internally focused mentation has been emphasized here, it is important to note that default mode activation has also been posited to reflect monitoring of the external environment (Gusnard and Raichle, 2001; Raichle et al., 2001). In the context of task fMRI, this could include waiting for upcoming task-relevant stimuli or attending to scanner noise and incidental light. Previous research indicates that older adults are vulnerable to distraction due to an inability to suppress processing of irrelevant environmental stimuli, including those related to the scanner environment (Stevens et al., 2008). Thus, the task-induced deactivation that we observed could also be related to suppression of external distractors which, as with suppression of internal distraction, would be expected to benefit their performance on an externally focused task.

The training-related increase in TID was specific to the encoding condition, underscoring the idea that the encoding phase may be more sensitive to the application of strategies and improvement. Supporting the importance of TID during encoding, Anticevic et al. (2010) reported greater DMN suppression for correct versus incorrect trials during encoding but not during later stages of a memory task (i.e., distracter and recognition probe phases). In another study, young adults showed greater DMN deactivation during encoding for scenes that were later remembered compared to those that were later forgotten (Chai et al., 2014). This suggests that DMN deactivation, and related suppression of distracting default mode processes, is especially important during initial formation of the memory traces.

*Post hoc* analyses revealed that the pattern of greater deactivation for the OLM group was apparent within the medial prefrontal cortex (mPFC), demonstrating a large effect size in comparison to the active control group across T2–T4. The mPFC exhibits particularly widespread connectivity within the DMN and may represent a network hub (Andrews-Hanna et al., 2014, 2010b). MPFC activity is linked to self-initiated stimulus-independent thought and emotional processing (McGuire et al., 1996; Binder et al., 1999; Gusnard et al., 2001). The mPFC may also play a unique role in suppression of task-irrelevant information. For instance, in young adults, mPFC deactivation during tests of working memory and attention was related to faster response times (Chadick and Gazzaley, 2011; Dang et al., 2013). Older adults exhibited less TID within the mPFC node of the DMN compared to young adults (Andrews-Hanna et al., 2007; Chadick et al., 2014), and among older adults reduced mPFC suppression was related to a greater impact of distraction on a working memory task (Chadick et al., 2014). In this context, our finding of increased training-related mPFC deactivation suggests that it is possible to ameliorate age-related decline in mPFC deactivation. Modulation of mPFC activity through learning was further demonstrated in a study of healthy older adults who showed a significant mPFC deactivation in response to the explicit instruction to apply a semantic encoding strategy; in this study a greater increase in deactivation was associated with greater strategic behavior (Balardin et al., 2015).

We observed a significant effect of sex in the longitudinal analysis, whereby men showed more dDMN deactivation during encoding than women across both the OLM and active control groups. There was no interaction with training group and importantly, there were no baseline (T1) differences in dDMN deactivation by sex. Sex differences in the DMN, particularly those regarding TID, have not been extensively studied. Some reports have described a higher degree of functional connectivity in women between the posterior cingulate/precuneus and prefrontal cortex (Bluhm et al., 2008; Tomasi and Volkow, 2012), while another found no sex differences in DMN connectivity (Weissman-Fogel et al., 2010). It is difficult to interpret our incidental finding that men exhibited greater TID from T2 to T4 across both training groups, but it may suggest that future training studies should more systematically examine sex differences in TID over time.

One final point about the interpretation of reduced TID in the elderly deserves mention here. The prevailing view in the literature is that reduced TID in older adults reflects a reduced ability to suppress default mode processes and reallocate resources toward the task at hand. However, potentially contradictory results arise from studies that separate deactivation related to successfully encoded items from that related to forgotten items (i.e., subsequent memory paradigm). Specifically, some studies have shown that older adults demonstrate less deactivation for remembered versus forgotten items, and in older adults successful encoding can be associated with less deactivation (Maillet and Schacter, 2016). These patterns, which differ from those observed in younger adults, raise the possibility that older adults are more reliant on the default mode than young adults when performing attention-demanding tasks (Maillet and Schacter, 2016). That is, greater engagement (i.e., reduced suppression) of the default network in older adults might reflect increased reliance on cognitive processes mediated by the DMN, such as drawing on prior knowledge, experience, and schemas accumulated over their longer lifespan (Turner and Spreng, 2015). If this interpretation is correct, DMN suppression could potentially hamper older adult performance, and it would call into question our interpretation that the training-related increase in TID is beneficial for older adults. Clearly, more research is needed to clarify the mechanisms underlying reduced TID in older adults and its functional implications, preferably involving the subsequent memory paradigm to address the discrepant patterns observed in old versus young adults.

### Limitations

Strengths of this study include the use of an active control training condition and multiple scanning sessions, but several limitations must be noted. First, it is unclear whether these results would generalize to the population of older adults, considering that our sample was relatively young (60–75 years), did not suffer from neurological or mental disorders, was highly educated, and had slightly above-average cognitive abilities as described in Zimmermann et al. (2016). Further, both groups performed the scanner task with approximately 79% accuracy at baseline, raising concerns about ceiling effects during the three subsequent sessions. There is also evidence that older adults demonstrate reduced TID relative to young adults primarily at higher levels of task demand (Persson et al., 2007; Park et al., 2010; Turner and Spreng, 2015). We cannot be sure that our task was challenging enough in this sense, but inclusion of a young adult comparison group and multiple task difficulty levels were not possible within the context of the current study.

Additionally, the atlas of Shirer et al. (2012), which we used to define the dorsal and ventral DMN subnetworks, is based on a sample of young adults. Grady et al. (2010) described a shrinkage of the extent of the DMN in terms of task-related brain activation patterns in older compared to younger adults, suggesting that a network definition based on a sample of young adults may not entirely apply to older adults. Mitigating this concern, data-driven parcellations of resting state data across different age cohorts reveal significant spatial overlap in large-scale brain systems across the adult lifespan (Han et al., 2018). In this context, we believe that the benefits of using the present atlas (i.e., standardization and comparability across studies) outweigh the concern about the young adult reference sample, but this caveat should still be considered when interpreting the results.

The block design employed in this study did not allow us to separate (de)activation related to successfully encoded items from that related to forgotten items. Known as the subsequent memory paradigm, this method is useful for elucidating neural processes related to successful learning (Daselaar et al., 2004; Maillet and Schacter, 2016). Further, the fact that our analysis calculated neural (de)activation levels across both successfully and unsuccessfully encoded items represents another possible explanation for the lack of an observed relationship with task improvement.

Finally, it is important to note that our ROI-based approach necessarily focuses on the DMN, and we cannot draw conclusions about the response to cognitive training in other regions or networks. We did not apply exploratory voxel-wise analysis because of considerations about statistical power and because of our *a priori* focus on the DMN. Nevertheless, we acknowledge that it could be useful for more exploratory types of analyses to apply the types of LMER models that we conducted here across all voxels of the brain (Madhyastha et al., 2018).

### Future Directions

Several directions for future research can be considered. First, the DMN does not function in isolation, but is thought to interact with other large-scale networks. Therefore, it might also be important to study training effects in the task-positive network that is preferentially active when individuals are engaged in attention-demanding tasks focused on the external environment (Fox et al., 2005). The task positive network may include a dorsal attention network as well as a frontoparietal control and a salience network (Seeley et al., 2007; Spreng, 2012). Some have argued that reduced DMN deactivation in the elderly is not reflective of DMN dysfunction *per se* but instead reflects a lower degree of flexible network interactivity, including greater coupling between DMN and frontoparietal/executive regions as task demands increase (Spreng and Schacter, 2012; Turner and Spreng, 2015). The salience network would also be of interest, considering that its disruption in aging is related to cognitive decline (Onoda et al., 2012) and that it exhibits increased connectivity to the DMN with increased age (Malagurski et al., 2020). Nevertheless, we found it necessary to first evaluate heterogeneity within the DMN before addressing between-network interactions. Future cognitive training studies may wish to examine dynamics across a wider range of networks, while also including multiple DMN subnetworks.

Another direction for future research involves examination of structural and functional connectivity as it relates to cognitive performance and training outcome. There is evidence that disruptions in white matter integrity may underlie aging-related decreases in DMN functional connectivity (Andrews-Hanna et al., 2007) as well as aging-related decreases in TID (Brown et al., 2018). Thus, the question arises as to whether training-related increases in TID observed in this study were related to changes in structural or functional connectivity.

### Conclusion

Our findings support the heterogeneity of the DMN, with the ventral DMN being activated during a memory task and the dorsal DMN being deactivated. Further, we report the novel finding that task-induced deactivation within the dorsal DMN was enhanced by process-based cognitive training compared to an active control training condition. Given reports of reduced DMN suppression in the elderly and negative functional consequences thereof, this finding may suggest a promising mechanism through which process-based cognitive training can enhance cognitive performance in the elderly. However, this conclusion is tempered by our incomplete understanding of the mechanism underlying TID reductions in older adults, as well as by the lack of an explicit association between increased TID and task improvement in the current study. Further research, including an examination of interactions with other networks and of associated structural and functional connectivity changes, could help to elucidate relevant mechanisms underlying training-related increases in dorsal DMN deactivation.

## Data Availability Statement

The data analyzed in this study is subject to the following licenses/restrictions: The R analysis code and the associated data tables will be made available by the authors upon request. The raw fMRI image data underlying this article are not publicly available. Requests to access these datasets should be directed to data@dynage.uzh.ch.

## Ethics Statement

The studies involving human participants were reviewed and approved by the Ethics Committee of the Canton of Zurich. The patients/participants provided their written informed consent to participate in this study.

## Author Contributions

AM: conceptualization, visualization, formal analysis, and writing – original draft preparation. BM: conceptualization, software, and writing – reviewing and editing. FL: software, data curation, and writing – reviewing and editing. SM: supervision, project administration, and writing – reviewing and editing. LJ: resources, funding acquisition, and writing – reviewing and editing. All authors contributed to the article and approved the submitted version.

## Conflict of Interest

The authors declare that the research was conducted in the absence of any commercial or financial relationships that could be construed as a potential conflict of interest.

## Footnotes

1 https://www.fil.ion.ucl.ac.uk/spm/software/spm12/
