# Supplementary material for: Object-Location Memory Training in Older Adults Leads to Greater Deactivation of the Dorsal Default Mode Network
Source: Front Hum Neurosci. 2021 Feb 26;15:623766. doi: 10.3389/fnhum.2021.623766 (PMC7952529; doi:10.3389/fnhum.2021.623766)
Supplement: Supplementary file 1 [file Table_1.DOCX]

Supplementary Material

## Supplementary Figures


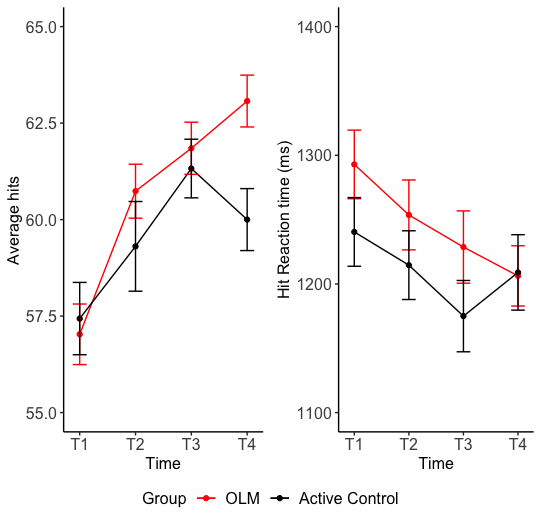


**Supplementary Figure 1.** Average hits and hit reaction time (ms) during the in-scanner object-location memory task. Error bars represent SEM. Average hits refers to the number of correct responses out of 72 items for each run, averaged across two runs. T1 = pre-training, T2 = training mid-point, T3 = training completion, T4 = 4-month follow-up.


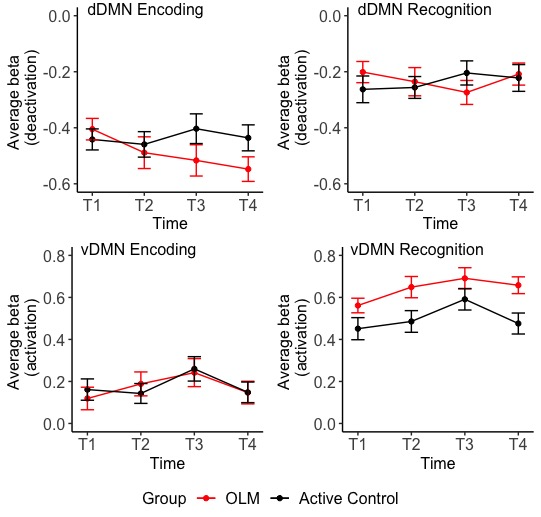


**Supplementary Figure 2.** Average beta values representing activation/deactivation at each time for dorsal and ventral DMN networks during both conditions of the in-scanner memory task. Contrasts represent encoding and recognition conditions relative to the visual fixation baseline condition. Error bars represent SEM. T1 = pre-training, T2 = training mid-point, T3 = training completion, T4 = 4-month follow-up.
